# A Case of Refractory Giant Porokeratosis, Treated With a New Treatment Approach Consisting of Ablative CO_2_ Laser and Methotrexate

**DOI:** 10.1002/ccr3.72963

**Published:** 2026-06-17

**Authors:** Ali Asilian, Mohammad Shoushtarizadeh, Sarah Seyedyousefi, Mohamad Beheshti, Malih Aminjavaheri

**Affiliations:** ^1^ Skin Diseases and Leishmaniasis Research Center Isfahan University of Medical Sciences Isfahan Iran

**Keywords:** CO_2_‐laser therapy, methotrexate, porokeratosis, treatment

## Abstract

Combined fractional CO_2_ laser + MTX may be an option for refractory GPK, with vigilant biopsy confirmation and malignancy surveillance.

## 1 Introduction

Porokeratosis (PK) is a term attributed to a rare group of epidermal keratinization disorders that can be either acquired or congenital, with higher prevalence in females [1, 2]. This condition was reported in different age groups, the youngest case reported being a 2‐year‐old male, and the eldest an 83‐year‐old male [3, 4, 5]. Multiple genetic mutations mostly in the mevalonate pathway were proposed as the etiology of this disease, which are primarily inherited in an autosomal dominant manner [1, 6, 7].

All subtypes of porokeratosis exhibit a characteristic histopathologic feature known as the cornoid lamella, which consists of a vertical column of parakeratotic cells within an otherwise orthokeratotic stratum corneum; this alongside with chronic inflammation is the hallmark of PK [7, 8, 9]. Clinically PK presents as annular plaques with an atrophic center and hyperkeratotic borders [7].

Disseminated superficial actinic porokeratosis is generally considered the most common clinical variant of porokeratosis, whereas porokeratosis of Mibelli represents the classic localized form, which is presumed to be associated with immunosuppression [1, 2, 10]. On the other hand, Giant porokeratosis (GPK) is recognized as a rare variant of PM and is clinically significant due to its association with squamous cell carcinoma (SCC) [9]. The prevalence of malignancy within PK is 7.5% with notable concern for GPK [11].

PK shares some molecular similarities with psoriasis. Therefore, some treatment approaches effective in psoriasis may also be beneficial for PK. Imiquimod cream has shown the best outcomes for PM among various treatment modalities. Protection from UV exposure and topical agents such as 5‐fluorouracil have greatly affected the treatment process. Topical statin therapy has also been shown to somewhat improve some of the lesions [2, 6, 12, 13]. However, no certain and specific treatment is currently available for PK, leading to multiple treatment approaches for affected patients [13].

In this study, we report a case of refractory GPK and her treatment course.

## 2 Case Presentation

### 2.1 Case History

A 53‐year‐old female patient, with a 21‐year history of GPK, presented to the dermatology clinic with the chief complaint of a refractory back lesion.

The lesion initially appeared as a small (with a diameter of 0.2 cm) cream‐colored and erythematous patch approximately 21 years prior to presentation and gradually enlarged. Over the past 14 years, it progressed in size and thickness, prompting repeated dermatologic evaluation. The diagnosis of giant porokeratosis was established following histopathological confirmation. She had complaints of an itching or burning sensation in times of intense sun exposure. Histological investigations were conducted and revealed epidermal acanthosis, moderate hyperkeratosis, and perivascular inflammatory infiltration. The findings mostly led to the diagnosis of PM. The patient had no family history of similar lesions, no other systemic disease, and no history of any medication use.

### 2.2 Differential Diagnosis, Investigations and Treatment

**FIGURE 1:**
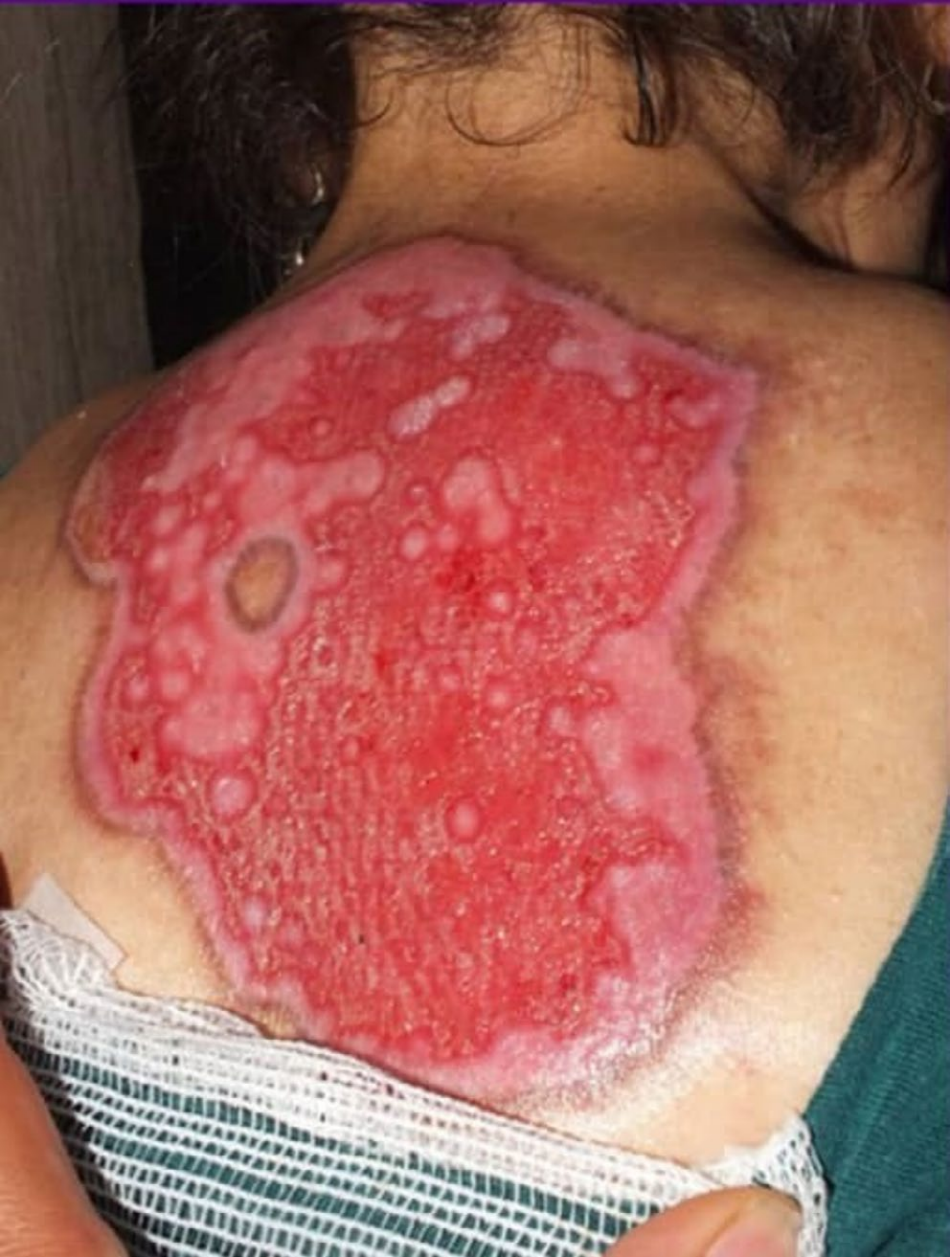
The GPK lesion on the back of the patient.

Clinical examination revealed a 5 × 4 cm exudative erosive erythematous plaque with irregular, raised hyperpigmented margins (Figure 1).

The lesion has expanded in size relative to the onset of the disease and also developed additional histopathological changes, including a mild increase in the granular layer and basal pigmentation, detection of a parakeratotic column within a hair follicle with localized atrophy, diffuse lymphohistiocytic and neutrophilic infiltration in the dermis, presence of macrophages containing melanin and eosinophils, and a small epidermal invagination with a parakeratotic column and epidermal ulceration.

Histopathologic examination was re‐reviewed by an experienced dermatopathologist. Sections showed epidermal invagination containing a column of parakeratosis compatible with a cornoid lamella, with focal loss/thinning of the granular layer beneath the parakeratotic column. The surrounding epidermis showed hyperkeratosis and mild acanthotic change. Importantly, features supporting discoid lupus erythematosus, such as interface/basal vacuolar degeneration, marked follicular plugging with interface change, epidermal atrophy, or dense periappendageal/perivascular lymphocytic infiltrate, were not identified. Features typical of pityriasis rubra pilaris or psoriasis‐like psoriasiform dermatitis, including broad regular acanthosis and elongated rete ridges, were also absent. Based on the clinicopathologic correlation, a diagnosis of giant porokeratosis was favored.

**FIGURE 2:**
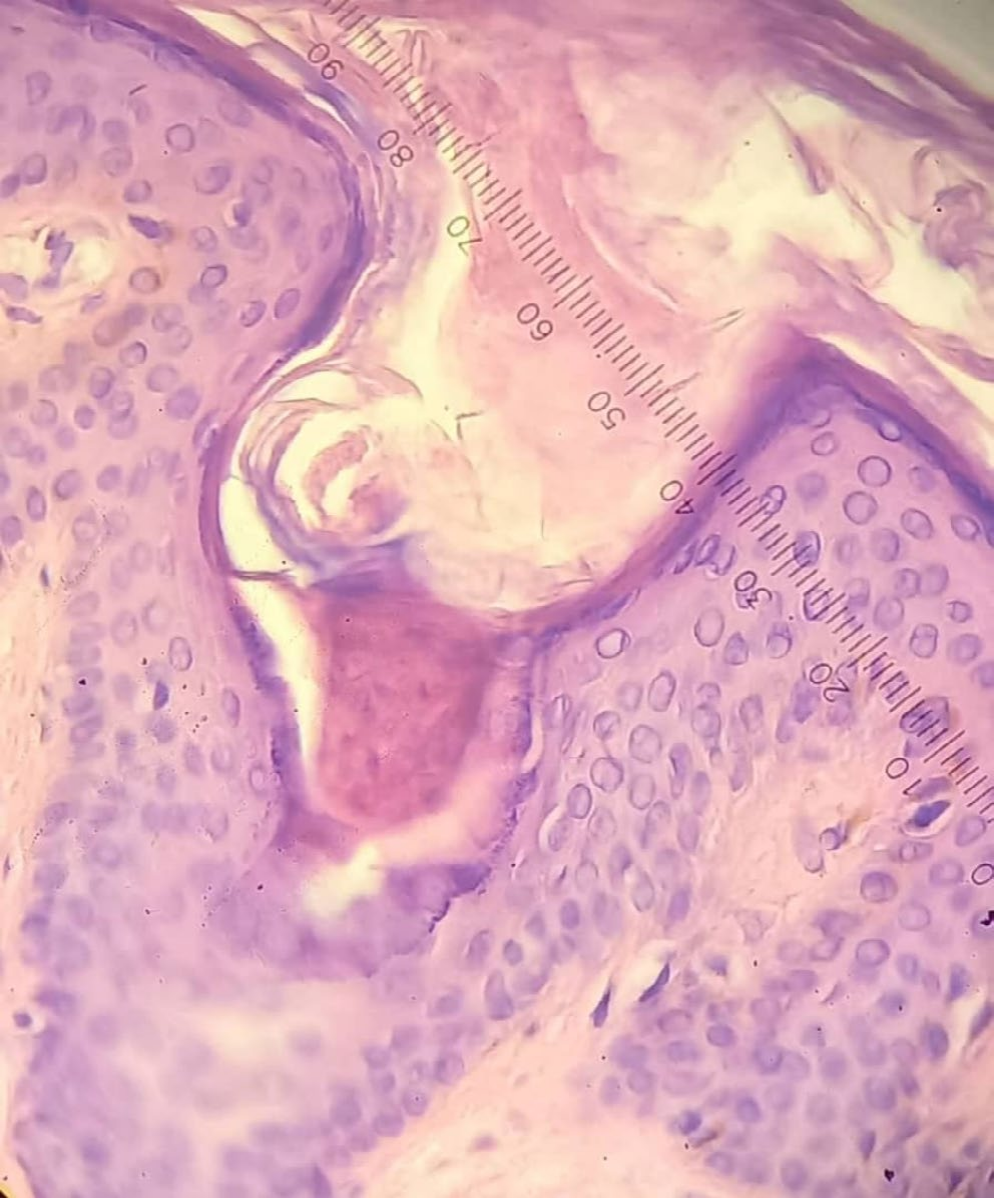
Porokeratosis Columns of parakeratosis arising from follicular unit (H&E 40×).

**FIGURE 3:**
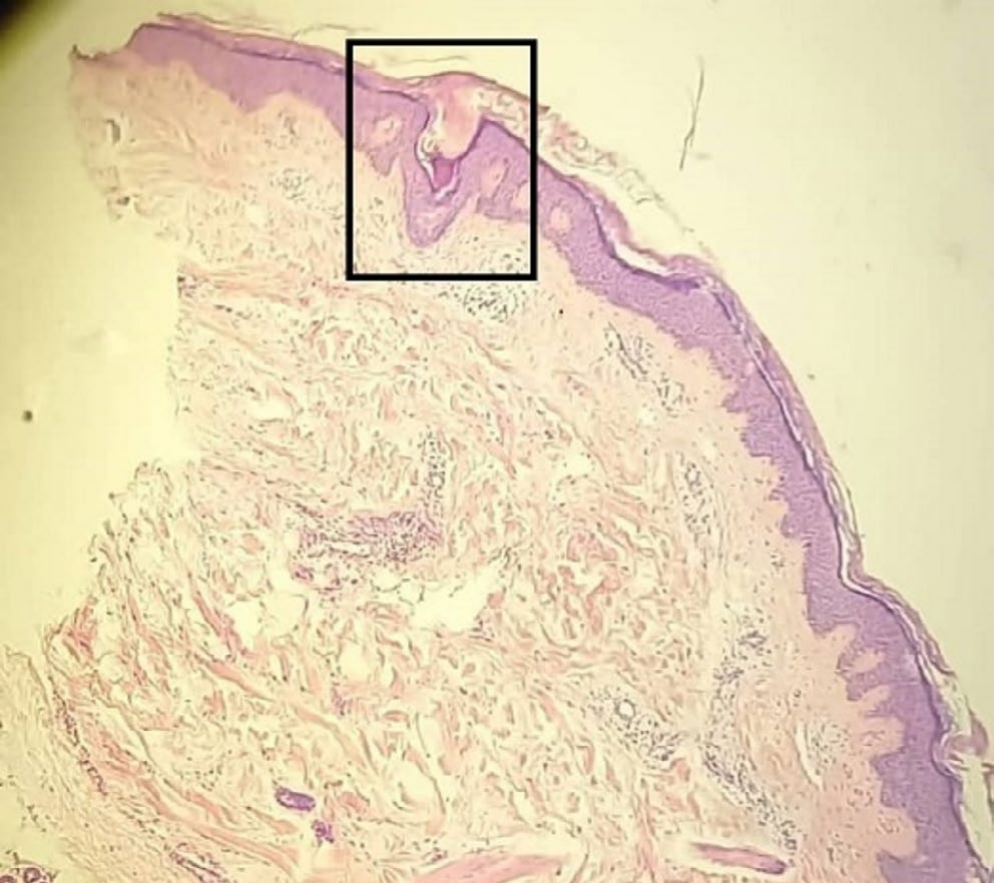
Porokeratosis. Note the coronoid lamella and hyper keratosis and hypogranolosis (black box) (H&E 4×).

Histopathological micrograph can be seen in Figures 2 and 3.

Due to these clinical and histopathological findings, and the size of the lesion, GPK diagnosis was established. Given the lesion's long duration, large size, chronic erosion/ulceration, and known malignant potential of long‐standing giant porokeratosis, malignant transformation to squamous cell carcinoma was clinically considered. Histopathologic assessment did not demonstrate definite invasive SCC in the examined sections.

The lesion was resistant to multiple unsuccessful treatment attempts. Over the course of her disease, the patient underwent a stepwise escalation of therapy in line with reported treatment strategies for porokeratosis. Initial management consisted of topical keratolytic and antiproliferative agents, including topical retinoids applied nightly for approximately 2 months, without any meaningful clinical improvement. This was followed by topical immunomodulatory therapy with tacrolimus 0.1% ointment twice daily and vitamin D analogs for a further 8 weeks, also without a complete and sustained response. Given the refractory nature of the lesion, immune response modifier therapy with imiquimod 5% cream three times weekly was subsequently attempted for approximately 4 months, resulting in only transient partial flattening of the hyperkeratotic border. Topical cytotoxic therapy with 5‐fluorouracil 5% cream applied once daily for 8 weeks and lesion‐directed cryotherapy (three sessions at monthly intervals) were also trialed, each producing only temporary improvement with recurrence at the margins. Owing to the extensive size of the lesion and concern for malignant potential, systemic retinoid therapy with oral acitretin (25 mg daily) was also administered for 6 months; however, this led to incomplete response and relapse after stopping the treatment, prompting consideration of alternative combination strategies.

We started the patient on an innovative combined approach. The selected therapeutic strategy involved combining fractional laser CO_2_ therapy with methotrexate, aiming for both structural remodeling of the affected skin and systemic disease control. This synergistic approach facilitated a progressive and sustained improvement in the patient's condition.

A fractional CO2 Laser was utilized to induce microthermal zones of controlled epidermal and dermal damage, promoting progressive skin remodeling. Laser parameters were carefully adjusted based on the lesion's size and depth to maximize efficacy while minimizing adverse effects.

Methotrexate as an immunosuppressive agent was applied to reduce abnormal keratinocyte proliferation and provide systemic disease control. The dosage was tailored to the patient's condition for optimal therapeutic impact.

Methotrexate was started at a dose of 10 mg taken orally once weekly, along with daily folic acid supplementation (1 mg per day) to reduce side effects. Before starting methotrexate, baseline laboratory tests including complete blood count, liver function tests, and renal function were obtained, and these were monitored every 4 weeks during treatment. In parallel, fractional CO_2_ laser treatment was applied to the lesion in three sessions at four‐week intervals. Each laser session was performed under topical anesthesia, with standard post‐procedure wound care using petrolatum ointment and strict sun protection. The treatment was well tolerated, with only mild and temporary redness after laser sessions. Progressive clinical improvement was observed after each session, and complete clinical clearance was achieved by the end of the treatment course. The details of this treatment regimen can be seen in Table 1.

**TABLE 1:** Details of the innovative combined treatment protocol.

Methotrexate dose and duration	10 mg orally once weekly for 12 weeks
Folate rescue	Folic acid 1 mg daily
Baseline laboratory tests	Complete blood count (CBC), liver function tests (LFTs), serum creatinine
Laboratory monitoring	CBC & LFTs every 4 weeks
Laser platform	Monthly fractional CO_2_ laser (10,600 nm) with an energy of 30–50 mJ, Density of 10–15, pulse duration of 0.8–1.2 ms, in 3 sessions
Perilaser care	Petrolatum ×7 days + sunscreen cream

**FIGURE 4:**
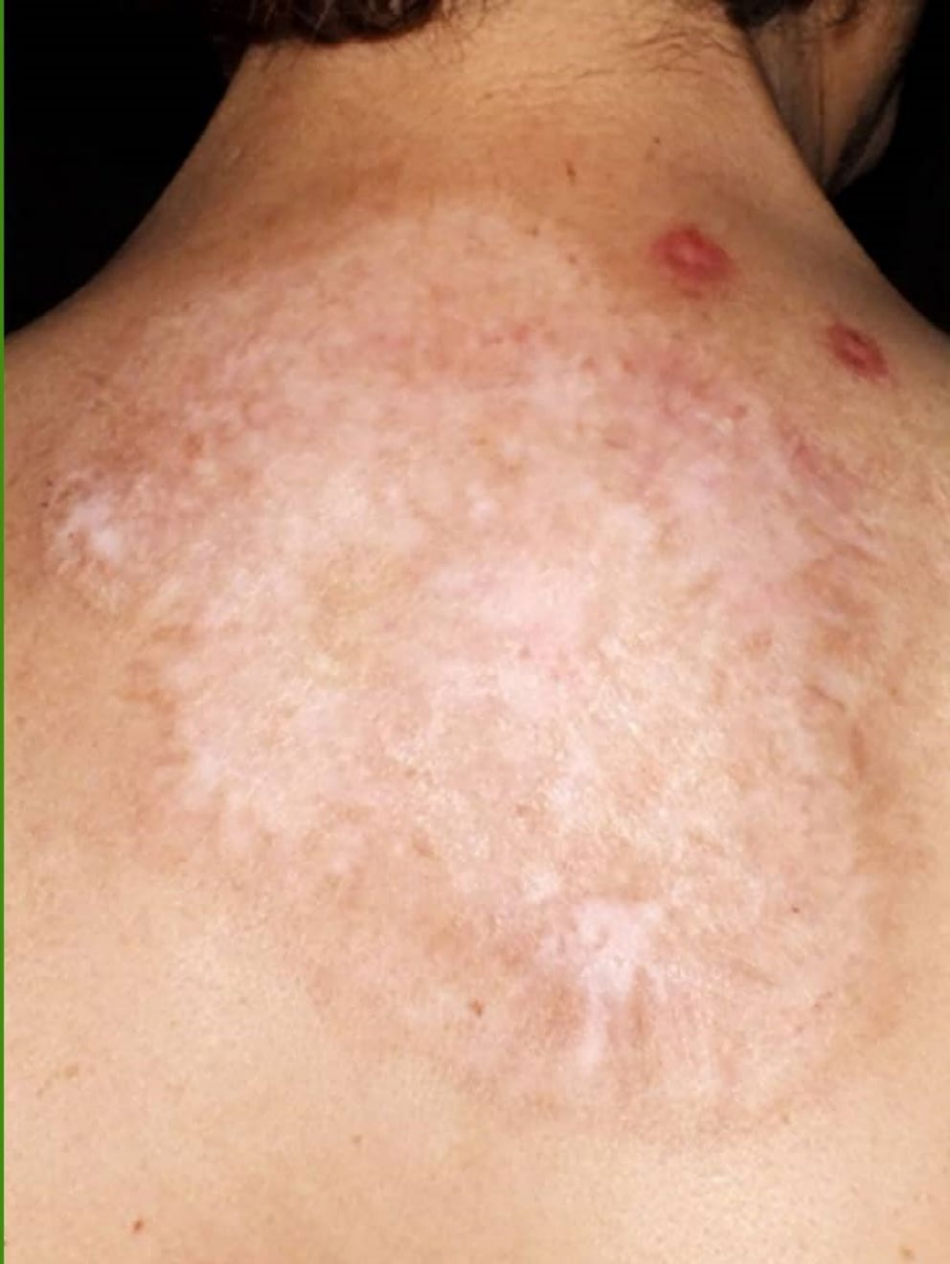
The GPK lesion after treatment.

Following complete clinical recovery, a 6‐year follow‐up was conducted with no signs of recurrence. Sustained remission was documented at 6 years with serial standardized photographs and absence of satellite lesions or marginal recurrence. This outcome underscores the long‐term efficacy and safety of the applied treatment strategy, highlighting its potential as a viable therapeutic option for similar cases (Figure 4).

## 3 Discussion

PM is a subtype of porokeratosis caused by keratinization disorder. GPK is considered a variant of PM, with a great concern for malignancy transformation [9, 14].

Several treatment modalities were proposed before, including surgical, topical, systemic, and procedural interventions [15]. Complete Surgical excision, particularly for SCC‐associated PK demonstrated remarkable success and a low recurrence rate [9, 16]. However, surgery might not always be the best approach, especially for extensive lesions or cosmetically sensitive areas [15, 16].

Topical agents are considered a primary approach for PK with a variable success rate. The most common treatment for PM is imiquimod 5% cream with reports of complete recoveries in many cases after a prolonged course of treatment [5, 13, 14, 15, 17, 18]. Imiquimod clears dysplastic keratinocytes due to activating an immune‐mediated inflammatory response; however, the scarring due to exacerbated inflammation is still a concern [15]. Also, imiquimod 5% cream might prevent keloid recurrences after surgery with variable success [19, 20]. 5‐fluorouracil also (5‐FU) showed notable efficacy, particularly in combination with other treatment approaches such as photodynamic therapy (PDT) [21]. The combination of 5‐FU and imiquimod showed inconsistent efficiency [5, 15].

More recently, Janus kinase inhibitors have been reported as potential therapeutic options in selected porokeratosis variants, particularly eruptive pruritic papular porokeratosis and porokeratosis ptychotropica. Case reports have described successful responses to agents such as abrocitinib and upadacitinib; however, evidence remains limited to isolated reports, and the role of JAK inhibition in giant porokeratosis has not yet been established [22, 23, 24].

Retinoids, both topical and systemic, have a role in modulating epidermal turnover and reducing carcinogenic potential and showed variable success. However, prolonged application is limited due to side effects [25].

Due to cholesterol reduction caused by genetic mutations, Cholesterol‐lovastatin ointment could improve skin barrier function and reduce apoptosis. However, this therapeutic approach is not sufficient on its own due to newer evidence demonstrating mevalonate accumulation as the primary role of PK and should be used alongside other treatment modalities [4, 17, 26].

Cryotherapy, laser therapy, and PDT showed improvement in the treatment process. PDT particularly combined with topical 5‐FU showed remarkable improvement and even complete recovery in some cases [15]. However, there is a high risk of scarring and pigmentation in long‐term cryotherapy and laser ablation, which limits their widespread use.

Nicholas A Ross et al. used fractional 1927‐nm laser for the treatment of patients with Disseminated superficial actinic porokeratosis which resulted in remarkable improvement and relief [27]. Rania Alakad et al. demonstrate no significant difference between methotrexate injection alone and a combination of methotrexate and fractional CO_2_ laser [28].

In the present case, fractional CO_2_ laser was selected to induce controlled remodeling of the hyperkeratotic and chronically altered lesion. Methotrexate was added empirically as an adjunctive systemic agent because of the refractory, chronic, inflamed nature of the lesion and its antiproliferative/immunomodulatory effects. We acknowledge that methotrexate is not an established standard therapy for porokeratosis, and the favorable outcome in this single case should be interpreted cautiously. Nevertheless, the sustained clinical remission over 6 years suggests that this combined approach may merit further investigation in carefully selected refractory cases.

## 4 Conclusion

This case highlights the therapeutic potential of combining fractional CO_2_ laser therapy with methotrexate for treating refractory giant porokeratosis (GPK), especially in patients unresponsive to conventional treatments. The synergistic effect of localized skin remodeling and systemic immunosuppression resulted in complete clinical remission with no recurrence over a 6‐year follow‐up period. This novel treatment strategy presents a promising and durable option for managing extensive or resistant porokeratosis lesions and warrants further investigation in larger clinical studies.

## Author Contributions

**Ali Asilian:** conceptualization. **Mohammad Shoushtarizadeh:** conceptualization, visualization, writing – original draft. **Sarah Seyedyousefi:** validation. **Mohamad Beheshti:** conceptualization, data curation. **Malih Aminjavaheri:** investigation, supervision, writing – review and editing.

## Funding

The authors have nothing to report.

## Disclosure

The authors have nothing to report.

## Ethics Statement

This study has obtained ethical approval from the Isfahan University of Medical Sciences.

## Consent

Written informed consent was obtained from the patient to publish this report in accordance with the journal's patient consent policy.

## Conflicts of Interest

The authors declare no conflicts of interest.

## Data Availability

All data used and analyzed during this study are available from the corresponding author upon request.
